# Positive Airway Pressure Therapy for Pediatric Obstructive Sleep Apnea

**DOI:** 10.3390/children8110979

**Published:** 2021-10-29

**Authors:** Kelly K. Hady, Caroline U. A. Okorie

**Affiliations:** 1 Department of Pediatrics, Valley Children’s Healthcare, Fresno, CA 93636, USA; khady@valleychildrens.org; 2 Department of Pediatrics, Stanford University School of Medicine, Stanford, CA 94305, USA

**Keywords:** pediatric sleep apnea, sleep disordered breathing, obstructive sleep apnea, positive airway pressure, auto-titrating continuous positive airway pressure

## Abstract

Pediatric obstructive sleep apnea syndrome (OSAS) is a disorder of breathing during sleep, characterized by intermittent or prolonged upper airway obstruction that can disrupt normal ventilation and/or sleep patterns. It can affect an estimated 2–4% of children worldwide. Untreated OSAS can have far reaching consequences on a child’s health, including low mood and concentration as well as metabolic derangements and pulmonary vascular disease. Most children are treated with surgical intervention (e.g., first-line therapy, adenotonsillectomy); however, for those for whom surgery is not indicated or desired, or for those with postoperative residual OSAS, positive airway pressure (PAP) therapy is often employed. PAP therapy can be used to relieve upper airway obstruction as well as aid in ventilation. PAP therapy is effective in treatment of OSAS in children and adults, although with pediatric patients, additional considerations and limitations exist. Active management and care for various considerations important to pediatric patients with OSAS can allow PAP to be an effective and safe therapy in this population.

## 1. Introduction

Pediatric obstructive sleep apnea (OSA) is a disorder of breathing during sleep, characterized by intermittent or prolonged complete or partial upper airway obstruction that can disrupt normal ventilation and/or sleep patterns. It is estimated to affect 2–4% of children worldwide [1]. OSA can be seen in otherwise healthy children; however, patients with certain underlying medical conditions, such as trisomy 21/Down syndrome, Prader–Willi syndrome, or Pierre Robin syndrome have a much higher incidence of OSA associated with their underlying condition [2,3,4]. Untreated OSA is associated with a variety of health and well-being concerns including impaired cognition, poor mood, poor growth, developmental delay, and attention deficit [5,6,7]. Prolonged or severe disease can be associated with cardiovascular complications, including systemic hypertension and pulmonary vascular disease [8,9]. Treatment of OSA depends upon the etiology of the obstruction as well as other clinical factors that need to be considered in the pediatric population. Treatment options can include surgical intervention, medication, orthodontic intervention, and positive airway pressure (PAP) therapy. PAP therapy is commonly used among adult patients and can be successfully used in pediatric patients when specific accommodations and interventions are considered. PAP therapy is effective when appropriate pressures and settings are selected and adherence barriers are overcome.

## 2. Diagnosing Obstructive Sleep Apnea

Untreated OSA in children is associated with several issues, including cardiovascular disease, low mood, metabolic derangements, and developmental issues. Because of the relatively high incidence of disease as well as the potential for significant clinical consequence, the American Academy of Pediatrics recommends that all children be screened for OSA with careful attention to patients with chronic snoring [10]. Signs and symptoms of OSA include mouth breathing, attention deficit, daytime sleepiness, frequent nighttime awakening, enuresis, and daytime crankiness. Patients who display these symptoms should be, at the minimum, screened for OSA, and if the suspicion exists, they should have testing with polysomnography [7]. A commonly considered symptom of OSA is chronic snoring, which is noted to be seen in 2.4–15.6% of the general population (with increased incidence among Black patients and patients with a history of prematurity) [11]. Although significant obstructive sleep apnea is a common symptom of OSA, it is important to note that some children can have the condition even in the absence of snoring [5,7,12]. Therefore, testing for OSA with in-lab polysomnography (PSG) is important in those patients without obvious *breathing* symptoms, but with other symptoms as mentioned previously (enuresis, attention disorder, disrupted sleep, or daytime sleepiness). The physical examination is an important part of the clinical screening for patients suspected of obstructive sleep apnea. In the clinic, providers can assess the nasal passages for evidence of obstruction (e.g., septal deviation or nasal turbinate hypertrophy) and the oropharynx for obstruction by the tonsils or tongue. In some clinical settings (typically in otolaryngology practices) awake laryngoscopy can be performed to visualize the adenoids. Additionally, drug-induced sleep endoscopy (DISE) may also have a place in the initial diagnosis, allowing for evaluation of surrounding deep structures to identify additional levels of obstruction. The DISE can inform the patient and provider of the potential efficacy of available treatment options, such as excision of deeper tissues or implantation of hypoglossal nerve stimulator.

While physical exam findings are certainly important, studies have shown that clinical evaluation alone may not sufficiently predict OSA in pediatric patients, increasing the importance of objective testing [7,13]. Other diagnostic studies, including pulse oximetry, and nap studies, may be helpful, but they are likely to underestimate sleep disordered breathing in children. Home sleep studies, often used in adults, are also suspected to underestimate the degree of sleep disordered breathing in children as they lack sleep EEG recording. However, progress continues to be made to make these tests more accessible to the pediatric population [14]. Recommended parameters and techniques (including sampling rates and filter settings) for a valid pediatric polysomnography have been well delineated in the American Academy of Sleep Medicine (AASM) scoring manual [15]. This manual and the recommendations must be used for AASM-accredited sleep labs. The recommendations are similar to that of adults with some exceptions, most notably the recommendation for carbon dioxide monitoring (often via end-tidal carbon dioxide monitoring or transcutaneous carbon dioxide monitoring). Additionally, a pediatric polysomnogram includes electroencephalogram (EEG), electromyogram (EMG) submental and bilateral tibial, electrocardiogram, nasal pressure, oronasal airflow, arterial oxygen saturation with pulse waveform, chest and abdominal wall motion (respiratory inductive plethysmography), body position sensor, video, and optional snoring microphone [15,16]. The polysomnogram can diagnose several sleep disorders including sleep disordered breathing, nocturnal seizures, movement disorders, and parasomnias; moreover, specific to OSA, it can objectively measure obstructive events and also detect associated changes in oxygenation or ventilation, as well as EEG arousal. It provides a comprehensive clinical picture of the various physiologic interactions and can help determine the character and severity of sleep disordered breathing [7,17]. When considering the striation of severity of OSA, the Apnea Hypopnea Index (AHI) is often used and takes a mix of obstructive apnea, central apnea, mixed apnea, and hypopnea events, and averages them per hour. The pediatric scale determines that an AHI > 1/h is considered abnormal with AHI between 1–5 considered mild, AHI > 5 and up to 10 considered moderate, and AHI > 10/h considered severe OSA [18,19]. It is important to note that the severity of the AHI does not always correlate with severity of daytime symptoms or impairment, and therefore treatment may be indicated even in patients with mild OSA [19]. Additionally, while PSG offers objective data to characterize the degree of dysfunction, some surgical interventions are performed even after a “negative” sleep study in the case of a symptomatic patient with adenoid or tonsillar hypertrophy. PSG is sometimes required in the United States in order to obtain insurance company approval for payment for procedures for some patients. Some have questioned the cost-effectiveness of requiring a PSG in every case where an adenotonsillectomy is considered for treatment of symptomatic OSA in the setting of adenoid or tonsillar hypertrophy [20].

## 3. Deciding to Use Positive Airway Pressure

As noted above, OSA is characterized by intermittent or prolonged partial or complete upper airway obstruction while sleeping. The cause of the upper airway obstruction can vary depending on the patient anatomy and underlying medical conditions. The most common cause of OSA in otherwise healthy children is adenotonsillar hypertrophy, and therefore surgical intervention (adenotonsillectomy) is noted to be the first-line therapy in most pediatric patients [7,21,22]. Despite adenotonsillectomy being the first line treatment, it is important to note that residual OSA may persist after surgical intervention, with increased risk of this among children with certain conditions, such as obesity, Down syndrome/trisomy 21, or Prader–Willi syndrome [22,23]. As mentioned above, in cases of residual OSA, undergoing a DISE may help the medical team and patient better understand the physiology of the residual OSA, which may in turn help inform whether additional targeted surgery is indicated. Importantly, there are a subset of patients for whom surgical intervention is not indicated, recommended, or desired. Specifically, surgical intervention may not be considered safe for patients with underlying cardiovascular, pulmonary, or neurologic disorders. In cases of residual OSA after surgery, and in cases where surgical intervention is not indicated, PAP therapy is effective and safe. PAP therapy has also been associated with important clinical benefits, including decreased insulin resistance, a reduction in cardiovascular disease risk, and an improvement in behavioral concerns in children [24,25,26,27,28,29]. Recognition of these benefits has increased over the years leading to increased prevalence of PAP therapy use.

## 4. What Is Positive Airway Pressure?

Positive airway pressure (PAP) therapy is often used in adults and has been shown to be an effective therapy for children with OSA [7,30]. PAP therapy involves a device that generates positive airway pressure at varying pressures, measured by centimeters of water pressure. The PAP machine needs to give pressures that exceed the critical closing pressure of the upper airway (pharynx) to maintain an open airway throughout the breathing cycle [7]. The different modes of PAP available will be described in more detail below. The effective treatment results from the pressure that is generated from the machine. The goals of PAP therapy include relieving the upper airway obstruction, reducing wake after sleep onset or associated oxygen desaturation. PAP therapy is also intended to normalize sleep architecture as well as improve quality of sleep and relieve daytime symptoms associated with poor sleep quality. PAP therapy can be used for the short term in patients awaiting interventions. It can also be used on a long-term basis, particularly in those patients for whom other interventions are not indicated or desired, or in patients with severe or syndromic craniofacial abnormalities.

## 5. Preparing for PAP Initiation

Upon deciding to initiate PAP therapy in the pediatric patient with OSA, it is important to review a few considerations. Specifically, pediatric patients with OSA should be evaluated for chronic nasal obstruction. If soft tissue obstruction is noted (i.e., turbinate hypertrophy or sinus congestion), treatment with nasal irrigation, intranasal steroids (such as fluticasone), antihistamines, or other systemic therapy (such as montelukast) should be considered and employed [31,32,33,34]. Intranasal fluticasone or triamcinolone are often used as initial therapy to shrink the adenoids and may provide significant relief if OSA is primarily due to adenoidal obstruction. Enabling nasal breathing can also help make PAP therapy better tolerated and effective [32,33,35,36,37].

Studies in adults demonstrate suboptimal adherence for a variety of reasons, and importantly, there is strong correlation between early adherence and long-term use (>6 months) [38]. Therefore, considering ways to set up your patient for success early in the process of PAP initiation is important for both adults and children as having an unpleasant initial exposure to CPAP is associated with prolonged treatment rejection [39,40]. It has been shown that unpleasant initial exposure to CPAP can increase the time of treatment rejection; therefore, approaching PAP therapy with intention and a plan is typically recommended. Of course, a shared decision-making approach is essential and can ultimately improve patient outcomes. First, understanding and addressing parental and patient pre-conceived notions and impressions and expectations of PAP therapy is important. This can help facilitate early debunking of disbeliefs or correction of incorrect impressions [39,40,41]. Next, considering interventions for PAP desensitization is important. It is thought that the most effective interventions typically involve a multifaceted, multidisciplinary approach to increasing tolerance of PAP therapy. Some training techniques already described in the literature include positive reinforcement (praising use of PAP), graduated exposure (incremental increases in PAP use), counter-conditioning (PAP therapy used at same time as other enjoyable activity, such as playing a video game), selective attention (caregiver praises positive behavior but ignores negative behavior), and escape prevention (redirecting children, preventing them from removing the PAP mask) [39,41,42]. Working to partner with caregivers of young children and with older children themselves is important to increase the chance of successful treatment with PAP therapy.

**Figure 1:**
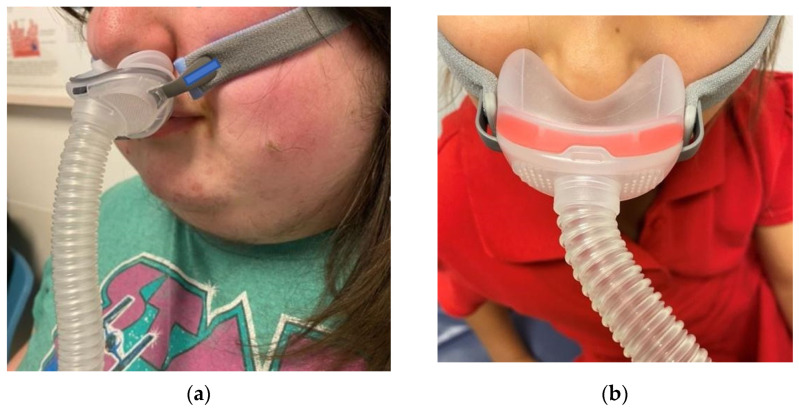
Nasal Pillows and Nasal Cradle Mask Examples (**a**) Nasal pillows PAP mask, sealing the nares. (**b**) Nasal cradle mask, sealing under the nares.

**Figure 2:**
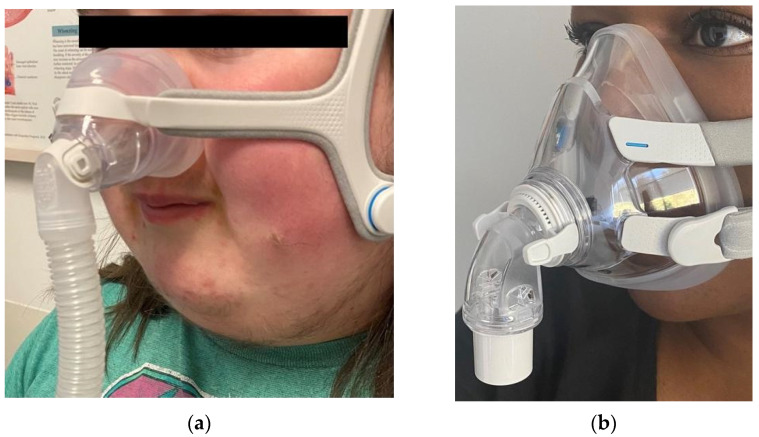
Nasal and Full Face Mask Examples (**a**) Nasal PAP mask, over the nose. (**b**) Full Face PAP mask covers nose and mouth.

When selecting an interface, it is important to find a good fit that offers comfort and minimal air leak for the patient. In terms of non-invasive PAP therapy options, interfaces include nasal pillows (which offer a seal at the nostrils, but do not cover the nose), nasal cradle masks (which fit under the nostrils, but do not cover the nose) (Figure 1), nasal masks (which can cover the entire nose), and full face masks (which cover the mouth and either the entire nose, or the nares) (Figure 2) or a total face mask (covers the entire face). Generally, nasal interfaces are preferred, as these may be more comfortable, allowing patients to speak, use a pacifier, or cough while wearing the PAP. Full face masks are often avoided in many cases if there is concern for possible aspiration after emesis or if the patient has copious oral secretions. Masks that can easily slip and cause obstruction of the nose should be avoided in young children. Studies observing adult preferences and adherence show that nasal masks tend to be preferred by most patients [43]. When considering a nasal mask, utilizing a gently applied chin strap in addition to the nasal mask can help avoid air leak through an open mouth.

When considering the PAP interface (or mask) there are a few special circumstances that need to be considered in the pediatric population. For instance, pediatric patients have growing orofacial bones and there is concern that long term use of nasal CPAP can contribute to midface hypoplasia related to pressure on the face [44,45]. Because of this risk, it is recommended that the individual risks/benefits of nasal PAP therapy be considered for each patient and that careful monitoring by an orthodontist is important to mitigate/minimize midface deformity [45,46]. Importantly, the changing faces of pediatric patients suggests the need for repeated evaluation of mask fit and comfort.

## 6. Choosing the Right Pressures/Settings

PAP therapy can be administered in a variety of modalities. The right modality depends on the patient’s specific needs. Fixed pressure continuous positive airway pressure (CPAP) is traditionally recommended in the pediatric population as it offers a stable level of positive pressure. The CPAP pressure is based on in lab polysomnography PAP titration. There are also auto-adjusting CPAP machines that use a proprietary algorithm to determine the occurrence of obstructive breathing events and increase (or decrease) the CPAP pressure accordingly. These may be helpful in patients who have OSA that varies in severity depending on body positioning and sleep stage. It can also adapt to weight changes or in the event of increased upper airway obstruction from intermittent upper airway inflammation caused by allergies or respiratory infections. PAP can also be delivered with dual pressure settings, referred to as bilevel positive airway pressure (BPAP). This modality offers a set pressure during inspiration (inspiratory positive airway pressure or IPAP) and another lower pressure during expiration (expiratory positive airway pressure or EPAP). BPAP can be used to treat OSA, especially if a patient requires higher pressures, is not tolerating the high CPAP pressure or if the patient has significant hypoventilation. Auto-adjusting BPAP is also available for those with sleep disordered breathing severity that may vary by sleeping position and sleep stage. More advanced support such as BPAP spontaneous-timed mode (or BPAP ST mode) is BPAP with a set respiratory rate to ensure a minimum number of breaths per minute are delivered. This setting can be helpful in patients with complex sleep apnea and patients with a central apnea component. Volume-assured pressure support (VAPS) mode offers an automatically titrating pressure support that is set to achieve a tidal volume goal that can be particularly helpful in patients with restrictive lung disease physiology secondary to obesity or neuromuscular weakness.

**Figure 3:**
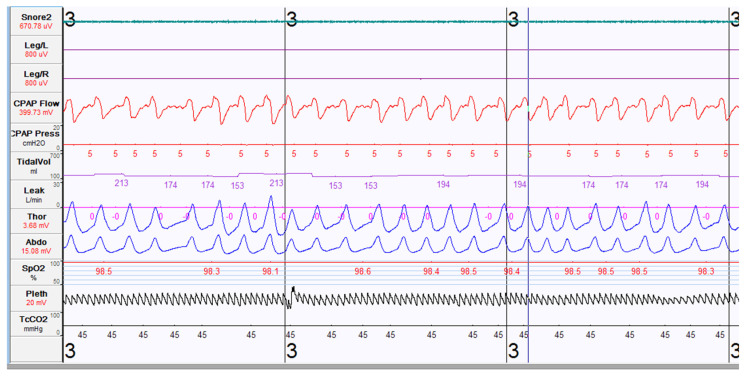
Polysomnography tracing during PAP titration study. Each tracing noted from top to bottom are as follows: snoring microphone, electromyogram of the bilateral tibia on the left and then the right leg, CPAP flow showing air flow, CPAP pressure setting of 5cmH20, tidal volume, air leak in L/min (currently at 0 L/min), chest and abdominal wall motion (respiratory inductive plethysmography), arterial oxygen saturation value and waveform, and transcutaneous carbon dioxide waveform (shown at 45 mmHg).

Once the decision to initiate PAP therapy has been made, and the discussion and consideration regarding the mask interface and potential desensitization needs have been reviewed, the provider needs to determine the correct setting and pressure most appropriate for the patient’s needs. Guidelines recommend that this be accomplished with an in-lab polysomnography with a PAP titration. A PAP titration is a polysomnography study during which the patient is trialed on different PAP therapies, while a technologist monitors in real time. Adjustments to the pressure or modality are made during the study, with the goal to resolve respiratory events, hypoxemia, or hypoventilation in a manner that allows the patient to sleep comfortably. An example of a PAP titration tracing is shown in Figure 3. Again, obstructive sleep apnea is a disorder of breathing and is worsened by conditions that further narrow or constrict the upper airway. Data have been mixed, but studies do suggest that, similar with adults, obstructive sleep apnea is more severe when pediatric patients sleep in the supine position [47,48]. This is consistent with sleep apnea pathophysiology seen in adults. Additionally, respiratory events are often more prevalent during the REM (rapid eye movement) stage of sleep. REM sleep is characterized by muscle atonia and irregular breathing rate. Specific to the upper airway, there is decreased tone of the upper airway, attributed to decreased genioglossus muscle tone, which can in turn cause increased upper airway obstruction [49]. Given these facts, the ideal polysomnogram with PAP titration should include resolution of respiratory symptoms even when the patient is supine, as well as in the REM sleep phase [50]. A titration is the best way to determine the most effective and tolerated pressure for the patient.

## 7. Monitoring Patients after PAP Initiation

**Figure 4:**
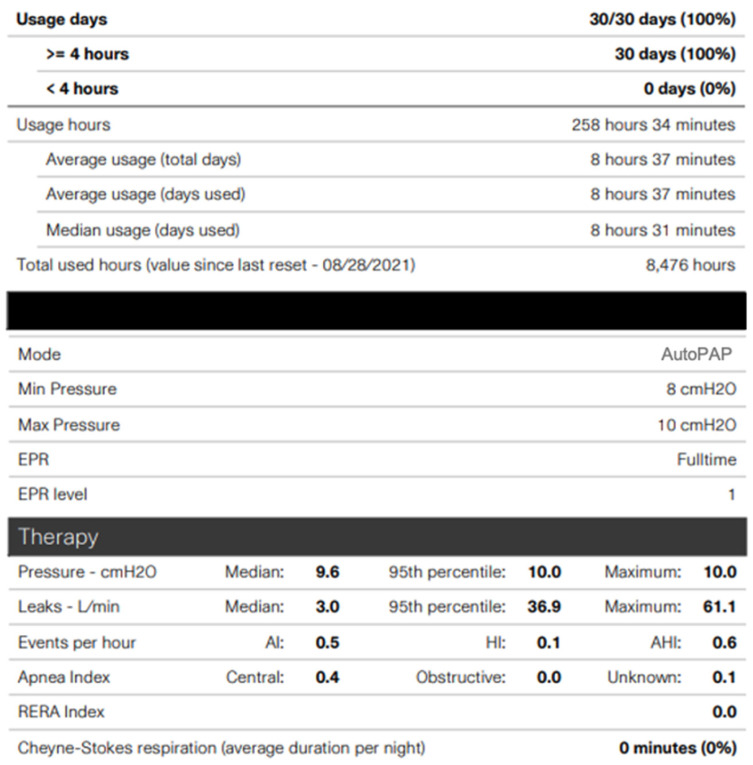
PAP adherence and therapy report of patient with OSA using an autoPAP machine. The data show usage over the last 30 days and provide average usage, pressure settings, and even leak. This report shows the patient has a low residual apnea hypopnea index (AHI) of 0.6/h and moderate air leak 36.9 L/min (ideal goal < 24 L/min).

**Figure 5:**
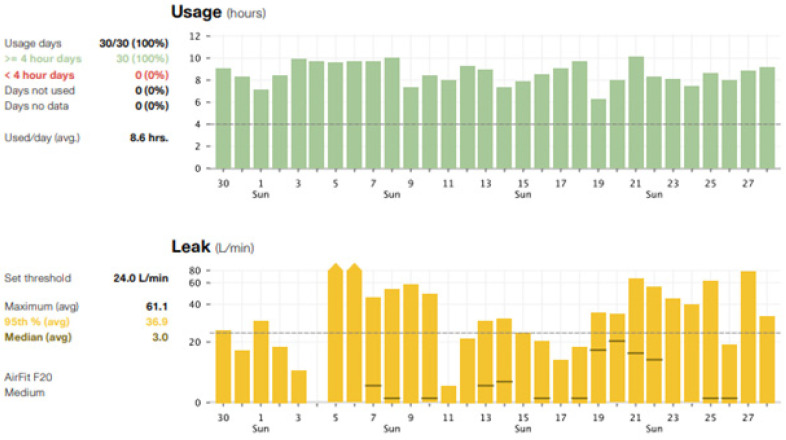
PAP adherence and therapy report of patient with OSA using an autoPAP machine. The data show usage over the last 30 days and average air leak. Monitoring use and air leak is important to determine if the patient is receiving adequate therapy and that mask fit remains appropriate.

Careful and close follow-up after PAP initiation is recommended and paramount to assure proper and safe medical therapy. The team managing the PAP therapy may vary depending on the clinical environment and resources. Typically, sleep medicine providers or pulmonologists manage PAP therapy; however, some practices employ skilled respiratory therapists to help manage as well. Providers should check in with patients and families to ensure proper equipment was received and that settings were accurately programmed. Having the families bring the PAP equipment to, at least, the first follow-up appointment can facilitate this and allow for any needed adjustments to occur quickly. Additionally, newer machines now enable cloud access to PAP therapy data, allowing the sleep medicine or pulmonology team to check for adherence and therapy efficacy remotely. When evaluating therapy data, the provider should pay special attention to the residual apnea hypopnea index (AHI) as measured by the machine, the measured air leak, the average or 95th percentile pressure (if autoPAP), and of course the timing and amount of use (Figure 4 and Figure 5). The AHI is not always accurate, and therefore these data should be evaluated in the context of other factors; however, ideally, the AHI should be <1/h. The measured air leak should be less than the “expected” air leak depending on the kind of PAP machine used. The manufacturer will note expected leak with each machine. The average or 95th percentile pressure in autoPAP is helpful to monitor if it seems the patient may require additional pressure, and the pressure maximum or minimum should be adjusted to allow any needed fluctuation in settings. Newer machines allow for these assessments and adjustments to be made remotely via a modem or cloud. This in combination with the expansion of telemedicine services can offer increased access to sleep medicine provider services. Manually checking and adjusting the machines is also possible as well.

As growing children may have evolving needs, having close follow-up is important. An initial or repeat PAP titration may be needed if large pressures/adjustments are needed or if the patient has changed signficantly. Patient changes can include pubertal growth, significant weight loss or gain (>10% of body weight), subsequent surgical or orthodontic intervention, or other changes in overall health status.

## 8. Positive Airway Pressure Adherence

Careful monitoring is important to assess adherence, tolerance, and effectiveness of therapy. Even among adult patients, adherence has been lower than desired, and many have struggled to better determine the factors and influences causing lower adherence rates. Within the adult literature, PAP adherence is defined as using the PAP device > 4 h/night on 70% of nights for 30 consecutive days, within the first 90 days of initiation [51]. One recent systematic review of PAP adherence for pediatric patients demonstrated adherence to be < 60% of patients with time of PAP use varying from 4.0 to 5.2 h of use a night [52]. Notably, many question whether 4 h is sufficient treatment in adults or pediatric patients. There are no data showing if this is a sufficient level of adherence.

Some patient characteristics that may influence PAP adherence have been identified. Specifically, younger age, female sex, and developmental delay are positive predictors of improved PAP adherence [52,53]. Patient family dynamics and demographics, rather than apnea severity, play a role in facilitating PAP adherence. Children with parents who take a proactive role in encouraging PAP adherence seemed to have increased PAP adherence, even among adolescents [54,55,56,57]. Reminders to use PAP as well as an authoritative style were all associated with increased PAP adherence, even among adolescents [54,58]. Children of parents who use PAP themselves may find therapy more acceptable [59]. This highlights the influence of the patient’s environment on their treatment outcome is notable [56]. The patient’s environment and home support group play an important role alongside support from the medical team. Ideally, sleep programs can include a multidisciplinary approach to ensure a collaborative and comprehensive integration of the device into a child’s daily life. In addition to the sleep physician/provider, a psychologist also assesses both the patient’s and caregiver’s attitudes and beliefs, identifying potential barriers and brainstorming possible solutions, thus thwarting adherence issues before they arise and during treatment. The Sleep Center at the Children’s Hospital of Philadelphia (CHOP) is an example of an intensive pediatric CPAP program that incorporates close and intensive follow-up and proactive management of patients and families using CPAP to achieve substantially higher follow up rates [39,42,60]. While education alone has been shown to have minimal effect on CPAP adherence, it is largely believed to influence a patient’s decision to both accept and continue treatment. Regular telemedicine visits can also provide reinforcement for those with distant or minimal caregiver support. Additionally, cognitive behavioral therapy, or CBT, has been used with some success to target psychosocial factors effectively [61].

There are many potential barriers to adherence for pediatric patients. The physical discomfort and/or fear of the machine often play a role. The desensitization interventions already mentioned include positive reinforcement, graduated exposure, counter-conditioning, selective attention, and escape prevention [39,41,42]. Additionally, optimizing air dynamics to provide sufficient opening of the posterior airway, while also maintaining a level of comfort that the patient can tolerate, is another one of the key factors in initial titration and adjustment of PAP settings. Finding the optimal PAP interface, as well as using comfort features such as humidification, heated tubing, pressure ramp, expiratory pressure relief (EPR), or A-flex/C-flex settings (which also offer a form of expiratory pressure drop) can all increase comfort and enhance adherence.

Addressing possible side effects of PAP early and effectively can also help improve adherence among pediatric patients. Skin irritation, specifically contact dermatitis, rash, redness, and even ulceration can all occur from use of the PAP mask and can be avoided/treated with careful monitoring and/or barrier interventions, topical treatments, or by alternating masks to allow skin time without contact [62,63]. Moreover, ensuring the mask is gently resting on the face (not tightly strapped down) is important to minimize pressure on both the skin and midface.

Irritation of the mucosa (rhinorrhea or nasal congestion) can result from high flows or cold air. Using a humidifier can help reduce the occurrence of inflammation from cool, dry air. Using a humidifier with heated tubing is often recommended to avoid “rainout” or condensation that occurs when warm air travels along a cool tube. The heated tubing (or an insulated tube) can avoid air cooling as it passes from the machine to the mask, thus avoiding condensation. If nasal congestion or rhinorrhea persist, the patient may benefit from treatments of chronic nasal congestion noted above, namely, nasal irrigation and/or intranasal steroids.

Other side effects can include aerophagia (swallowing of air into the stomach) that can cause bloating and discomfort. Treatment or avoidance of aerophagia can include reducing the pressure level or transitioning from CPAP to bilevel PAP [62,64].

## 9. Auto-Titration CPAP in Pediatric Patients

In recent years, PAP companies have worked to determine technology and settings designed to improve adherence and efficacy. Autotitrating CPAP, or autoPAP, works by monitoring overnight airflow and utilizing specialized algorithms to provide variable pressure delivery specific to the patient. Although no studies have shown a clinically significant difference in compliance and quality of life compared to fixed-pressure CPAP, autoPAP has been widely used and well-documented in use for adult patients with obstructive sleep apnea, most notably for the initiation phase of therapy. It also has been indicated in patients who may have OSA that differs in severity, depending on the body position or sleep stage (e.g., worse in REM sleep). Limited data have been obtained regarding the use of autoPAP in children; however, multiple studies have found autoPAP to be a safe and effective means of initiating CPAP in children, with automatically derived treatment pressures similar to those from pressures determined during PAP titration studies using polysomnography [53,61,65]. The utility of autoPAP in patients has been raised again during the time of the SARS-CoV-2 pandemic. To avoid exposure of patients and healthcare workers, telemedicine became more widely used to continue patient evaluation and treatment, while many sleep centers temporarily halted in-lab polysomnography [66,67]. Forced reliance upon creative methods to initiate treatment may increase consideration of autoPAP. Considering initiation of autoPAP in older pediatric patients without significant cardiopulmonary disease but with access to sleep medicine services may be a promising approach to increase access and avoid delay of care. As the need for convenient and efficient medical care becomes more prominent, it can be speculated that autoPAP will be used more and more for pediatric OSA therapy.

## 10. Positive Airway Pressure Cessation

Given the potential for changing anatomy in children, considering when it is time for PAP cessation is important as well. PAP can be used for the long term, and some children may require support for months to years; however, patient needs may change over time. Patients should be carefully monitored for change in symptoms and changing needs [68]. Again, a growth spurt, surgical or orthodontia intervention or significant weight loss may reduce upper airway obstruction and associated sleep disordered breathing. A repeat polysomnography can help evaluate for continued presence of obstructive sleep apnea or hypoxemia. There are no well-established guidelines for the general pediatric population for frequency of PSG monitoring, although some guidance exists for specific congenital medical conditions such as Prader–Willi syndrome, trisomy 21/Down syndrome, and achondroplasia. Resolution of events and/or symptoms can indicate the need for repeat polysomnography and/or the ability to safely stop PAP treatment.

## 11. In Conclusions

Obstructive sleep apnea syndrome is one of the most prevalent sleep breathing disorders and can affect children and adults alike. Early diagnosis and treatment are important to avoid short-term and long-term effects on overall health and quality of life. PAP therapy is effective in treatment of OSA in children and adults, although with pediatric patients, it is important to consider additional factors and limitations of treatment. Active management and attention to various considerations, including facilitating desensitization, monitoring for specific side effects, and frequently adjusting to the changing needs of the growing pediatric patient are paramount to ensuring safe and effective use of PAP therapy for children with obstructive sleep apnea syndrome.
